# CEACAM1 recognition by bacterial pathogens is species-specific

**DOI:** 10.1186/1471-2180-10-117

**Published:** 2010-04-20

**Authors:** Maike Voges, Verena Bachmann, Robert Kammerer, Uri Gophna, Christof R Hauck

**Affiliations:** 1 Lehrstuhl für Zellbiologie, Universität Konstanz, Mailbox X908, 78457 Konstanz, Germany; 2 Konstanz Research School Chemical Biology, Universität Konstanz, 78457 Konstanz, Germany; 3 Institute of Immunology, Friedrich-Löffler-Institut, Tübingen, Germany; 4 Department of Molecular Microbiology and Biotechnology, Tel Aviv University, Tel Aviv, Israel

## Abstract

**Background:**

Carcinoembryonic antigen-related cell adhesion molecule 1 (CEACAM1), an immunoglobulin (Ig)-related glycoprotein, serves as cellular receptor for a variety of Gram-negative bacterial pathogens associated with the human mucosa. In particular, *Neisseria gonorrhoeae*, *N. meningitidis*, *Moraxella catarrhalis*, and *Haemophilus influenzae *possess well-characterized CEACAM1-binding adhesins. CEACAM1 is typically involved in cell-cell attachment, epithelial differentiation, neovascularisation and regulation of T-cell proliferation, and is one of the few CEACAM family members with homologues in different mammalian lineages. However, it is unknown whether bacterial adhesins of human pathogens can recognize CEACAM1 orthologues from other mammals.

**Results:**

Sequence comparisons of the amino-terminal Ig-variable-like domain of CEACAM1 reveal that the highest sequence divergence between human, murine, canine and bovine orthologues is found in the β-strands comprising the bacteria-binding CC'FG-face of the Ig-fold. Using GFP-tagged, soluble amino-terminal domains of CEACAM1, we demonstrate that bacterial pathogens selectively associate with human, but not other mammalian CEACAM1 orthologues. Whereas full-length human CEACAM1 can mediate internalization of *Neisseria gonorrhoeae *in transfected cells, murine CEACAM1 fails to support bacterial internalization, demonstrating that the sequence divergence of CEACAM1 orthologues has functional consequences with regard to bacterial recognition and cellular invasion.

**Conclusions:**

Our results establish the selective interaction of several human-restricted bacterial pathogens with human CEACAM1 and suggest that co-evolution of microbial adhesins with their corresponding receptors on mammalian cells contributes to the limited host range of these highly adapted infectious agents.

## Background

The immunoglobulin (Ig) superfamily contains a large number of receptors that serve as cell adhesion molecules (CAMs) mediating homotypic cell-cell-adhesion in multicellular animals. One group of mammalian IgCAMs is named according to the carcinoembryonic antigen (CEA), a tumor marker used in the surveillance of colon cancer [1]. Interestingly, CEA is only known from primates, where it is expressed by mucosal epithelial cells. Similar to CEA, most other CEA-related CAMs (CEACAMs) are restricted to specific mammalian lineages, and only a few CEACAMs, such as CEACAM1 or CEACAM16-20, have orthologues in distantly related mammals [2-4]. Accordingly, sequence comparisons based on published genome data have provided evidence that CEACAMs have independently diversified in each mammalian order [3,5].

In humans, CEACAM1 is the target of several Gram-negative commensal and pathogenic bacteria that inhabit the nasopharyngeal, intestinal, or urogenital mucosa. In particular, *Neisseria gonorrhoeae*, *N. lactamica*, *N. meningitidis*, *N. subflava*, *Haemophilus influenzae*, *Moraxella catarrhalis*, and *Escherichia coli *strains have been found to associate with the protein core or carbohydrate structures of this glycoprotein [6-11]. These bacterial species utilize distinct surface proteins (adhesins) to engage CEACAMs. For example, the neisserial colony opacity associated (Opa) proteins allow gonococci and meningococci to bind several CEACAM family members including CEACAM1, CEA, and CEACAM6, which are expressed on the apical surface of mucosal epithelial cells. Opa proteins are integral outer membrane proteins with 8 transmembrane β-strands and 4 small extracellular loops, with the central loops participating in CEACAM recognition [12]. Opa-like proteins with a similar β-barrel structure are also found in commensal *Neisseria *species and can mediate the association with CEACAM1 [11]. In addition, several typeable and non-typeable strains of *Haemophilus influenzae*, a species that shares the mucosal habitat and lifestyle of *Neisseria*, can engage CEACAM1 via their outer membrane protein P5 [9]. Another inhabitant of the human oro-pharyngeal mucosa, *Moraxella catarrhalis*, can bind via the UspA1 surface protein to the N-terminal domain of CEACAMs [10]. UspA1 belongs to the family of trimeric autotransporter or oligomeric coiled-coil adhesin (Oca) family. The prototype of the Oca family is the adhesin YadA of enteropathogenic *Yersiniae *that has a lollipop structure with a head group, an extended coiled-coil stalk region and a membrane anchor domain [13]. The mature trimeric UspA1 with a size of about 250 - 300 kDa protrudes up to 60 nm from the bacterial surface and is therefore completely distinct from membrane-embedded neisserial Opa proteins or the *Haemophilus *protein P5 [13]. Surprisingly, CEACAM recognition by the *Moraxella *UspA1 is mediated by a short sequence within the stalk region requiring a bend conformation of the UspA1 extracellular domain to accommodate CEACAM1 binding [14]. *Moraxella *strains lacking this peptide sequence within their stalk region fail to bind to CEACAMs [15].

The striking convergent evolution of structurally distinct adhesive proteins to engage CEACAM1 suggests that this binding is important during the life cycle of these bacteria. As all CEACAM-binding bacteria greatly differ in their pathogenic potential, but share the same ecological niche, it is highly likely that CEACAM-binding promotes colonization of the mucosa. Indeed, in vitro experiments have suggested that CEACAM-binding is not only a means to firmly attach to the host cell surface, but also suppresses the detachment of infected epithelial cells [16]. CEACAM-targeting bacterial adhesins might therefore represent colonization factors that promote the ability of bacteria to establish a firm foothold in their ecological niche. Whether this specialization is also a determinant of the host range of these bacterial pathogens is not known. Though bacterial species expressing CEACAM-binding adhesive proteins are in most cases human-specific, and have no other natural host organism, it has not been experimentally tested whether their adhesins selectively recognize human CEACAMs or can also bind to orthologues from other mammalian species.

In the present study, we analysed the binding of CEACAM1 orthologues from several mammals to bacterial pathogens with distinct adhesive proteins. In particular, we tested Opa protein-expressing *N. gonorrhoeae *and *N. meningitidis *as well as UspA1-expressing *M. catarrhalis *for their ability to recognize CEACAM1 homologues of human, murine, canine or bovine origin. Biochemical binding studies clearly demonstrate that these bacteria selectively interact with human CEACAM1. Furthermore, analyses of bacterial internalization show that the observed amino acid changes in the amino-terminal domain of mammalian CEACAM1 orthologues have clear-cut functional consequences. Accordingly, our data not only demonstrate that bacterial adhesins have co-evolved with the receptor molecules of their mammalian host, but also support the view that the diversification of CEACAMs in mammalian lineages is a pathogen-driven process.

## Methods

### Amino acid sequence alignment

For the amino acid sequence alignment of the N-terminal domains of CEACAM1 following sequences were used: human CEACAM1 (hCEA1, NM_001712), murine CEACAM1a (mCEA1, BC016891), canine CEACAM1 (cCEA1, NM_001097557.1), bovine CEACAM1 (bCEA1, AY345129), bovine CEACAM1 isoform b (bCEA1b, AY487418). The alignment was performed using CLUSTALW.

### Cell culture and transfection

The human embryonic kidney cell line 293T (293 cells) was cultured in Dulbecco's modified Eagle's medium (DMEM) containing 10% calf serum at 37°C in 5% CO_2 _and subcultured every second to third day. 293T cells were transfected by calcium-phosphate coprecipitation using 5 - 8 μg of plasmid DNA for each 10 cm culture dish.

### Bacteria and infection

Opa_52_-expressing (Opa_CEA_), non-piliated *N. gonorrhoeae *MS11-B2.1 (strain N309), and non-piliated, non-opaque gonococci MS11-B2.1 (strain N302) were kindly provided by T.F. Meyer (Max-Planck Institut für Infektionsbiologie, Berlin, Germany) and were cultured as described previously [17]. Opa-expressing, non-encapsulated *N. meningitidis *(SiaD mutant of strain MC58) was obtained from Matthias Frosch (Institut für Hygiene und Mikrobiologie, Universität Würzburg, Germany). *M. catarrhalis *strain ATCC 25238 was obtained from DSMZ (Braunschweig, Germany). Both *Moraxella *and *Neisseriae *were grown on GC agar plates (Difco BRL, Paisley, UK) supplemented with vitamins at 37°C, 5% CO_2 _and subcultured daily. For infection, bacteria were suspended in DMEM and the optical density of the suspension was used to estimate the number of the microorganisms according to a standard curve generated for each strain.

### Recombinant plasmid constructs

Mammalian expression plasmids encoding GFP-tagged human CEACAM1-4L (hCEACAM1-4L), human CEACAM1-4S, and the amino-terminal domain of human CEACAM1 (hCEA1-N) were described previously [18,19]. Murine CEACAM1-4S was constructed by amplifying the full-length cDNA of murine CEACAM1-4S (clone BF584691; ImaGenes, Berlin, Germany) with primers mCEACAM1-sense 5'-GAAGTTATCAGTCGACATGGAGCTGGCCTCAGCAC-3' and mCEACAM1-anti 5'-ATGGTCTAGAAAGCTTCCGCCAGACTTCCTGG-3'. The amino-terminal domain of murine CEACAM1 was amplified with primers mCEACAM1-sense and mCEACAM1-N-anti 5'-ATGGTCTAGAAAGCTTGGGTGTACATGAAATCGC-3'. The N-terminal domains of bovine CEACAM1 isoforms a and b as well as canine CEACAM1 were amplified from full-length cDNA using primers bovine CEACAM1abN for 5'-GAAGTTATCAGTCGACATGGGGACCCCCTCAG-3', bovine CEACAM1aN rev 5'-ATGGGTCTAGAAAGCTTGGGAGTATGTGGAGGTGTCCAG-3', bovine CEACAM1bN rev 5'-ATGGTCTAGAAAGCTTTGGAGTACGTGGAGGTGTCC-3', canine CEACAM1N for 5'-GAAGTTATCAGTCGACATGGAGCCCCCCTCG-3' and canine CEACAM1N rev 5'-ATGGTCTAGAAAGCTTGGGAATACTTGGAGCTGTCC-3'. All the resulting PCR fragments were cloned into pDNR-Dual using the In-Fusion PCR Cloning Kit (Clontech, Mountain View, CA) and transferred by Cre-mediated recombination into pLPS-3'EGFP (Clontech) resulting in GFP fused to the carboxy-terminus of the expressed proteins. Full-length human CEACAM1-4S and murine CEACAM1-4S were also transferred from pDNR-Dual into pLPS3'mCerulean resulting in mCerulean fused to the carboxy-terminus of the expressed proteins. pLPS3'mCerulean was generated by replacing the GFP coding sequence in pLPS3'EGFP with the cDNA encoding mCerulean [20] generously provided by D.W. Piston (Department of Molecular Physiology and Biophysics, Vanderbilt University, Nashville, Tennessee, USA).

### Cell lysis and Western blotting

Cell lysis and Western blotting were performed as described [17] using a rabbit polyclonal antibody against His-tagged GFP (produced at the animal core facility; University of Konstanz) or a monoclonal antibody against Opa proteins (clone 4B2/C11; generous gift of Marc Achtman, MPI für Infektionsbiologie, Berlin, Germany). Secondary antibodies were from Jackson ImmunoResearch (West Grove, PA).

### Binding studies of the different pathogens

Binding studies of the different pathogens with the soluble N-terminal domains of human, murine, bovine and canine CEACAM1 were performed as described [19]. Briefly, 4 × 10^7 ^bacteria were added to CEACAM1-N-domain-containing cell culture supernatants in a total volume of 1 ml and incubated for 30 min. After four washing steps, the samples were analysed on a LSR II flow cytometer (BD Bioscience, Heidelberg, Germany) by gating on the bacteria (based on forward and sideward scatter) and measuring bacteria-associated GFP fluorescence. In each case, 10 000 events per sample were obtained.

### Gentamicin protection assay

Gentamicin protection assays were conducted as described [17]. Briefly, 5 × 10^5 ^293 cells were seeded in 24-well plates coated with 10 μg/ml poly-L-lysine. Cells were infected with 30 bacteria/cell (MOI 30) for two hours. Then, the medium was replaced with DMEM containing 50 μg/ml gentamicin. After 45 min of incubation in gentamicin-containing medium, cells were lysed by the addition of 1% saponin in PBS for 10 min. Suitable dilutions were plated in triplicates on GC agar to determine the number of recovered viable bacteria.

### Flow cytometry invasion assay

Bacterial uptake by transfected 293 cells was analysed by flow cytometry as described [21]. Prior to infection, bacteria were labelled with 0.2 μg/ml 5-(6)-carboxyfluorescein-succinylester (fluorescein; Invitrogen-Molecular Probes, Karlsruhe, Germany) in PBS at 37°C for 30 min. Cells were infected with labelled bacteria at an MOI of 30 for 2 h. After infection, cells were washed with PBS and the samples were analysed on a LSR II flow cytometer (BD Bioscience) by gating on the cells based on forward and sideward scatter. Cell-associated fluorescein fluorescence was measured in the presence of 2 mg/ml trypan blue to quench fluorescence of extracellular bacteria and to selectively detect the fluorescence derived from intracellular bacteria. The percentage of fluorescein-positive cells was multiplied by the mean fluorescence intensity of the sample to obtain an estimate of the total number of internalized bacteria (uptake index). In each sample 10,000 cells were counted.

### Immunofluorescence staining

293 cells transfected with the indicated constructs were seeded onto poly-L-lysine- and fibronectin-coated (10 μg/ml and 4 μg/ml, respectively, in PBS) coverslips in 24-well plates. Cells were infected for 2 h with 5-(and-6)-carboxytetramethylrhodamine-succinimidyl- and biotin-labelled Opa_CEA_-expressing *N. gonorrhoeae *at an MOI of 20 essentially as described [22]. To discriminate between extracellular and intracellular bacteria, infected samples were fixed with 4% paraformaldehyde in PBS and washed three times with PBS, prior to incubation in blocking buffer (PBS, 10% FCS) for 15 min. Extracellular bacteria were stained with AlexaFluor647-streptavidin (Invitrogen, Karlsruhe, Germany) diluted 1:100 in blocking buffer for 1 h. Following three washes, samples were embedded in mounting medium (Dako, Glastrup, DK).

Samples were viewed with a TCS SP5 laser scanning confocal microscope (Leica Microsystems, Wetzlar, Germany) using a 63×, 1.3 NA Plan Neofluar oil-immersion objective. Fluorescence signals of triple-labelled specimens were serially recorded to avoid bleed-through. Images were digitally processed with NIH ImageJ and merged to yield pseudo-coloured pictures.

## Results

### Mammalian CEACAM1 orthologues show conserved as well as divergent regions in their amino-terminal domains

**Figure 1:**
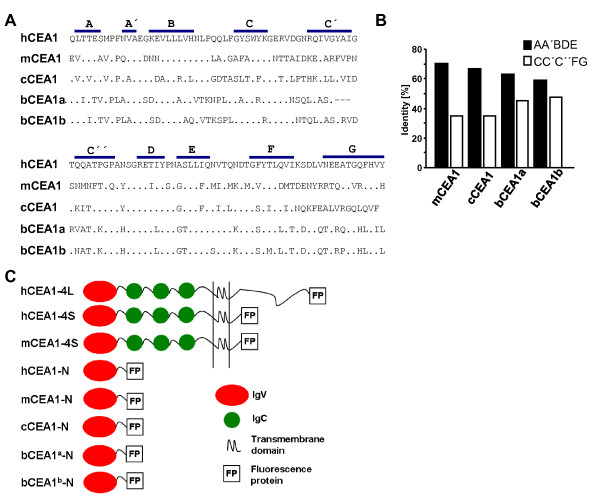
**Amino acid sequence alignment and expression of soluble CEACAM1 proteins of different mammals**. (**A) **Amino acid sequence alignment of the N-terminal domains of human, murine, bovine and canine CEACAM1 proteins. The following sequences were used: human CEACAM1 (hCEA1, NM_001712), murine CEACAM1a (mCEA1, BC016891), canine CEACAM1 (cCEA1, NM_001097557.1), bovine CEACAM1 (bCEA1, AY345129), bovine CEACAM1 isoform b (bCEA1b, AY487418). Amino acids identical to the human CEACAM1 sequence are indicated by dots. The characteristic beta-strands of the Ig variable-like domain are marked by blue lines and letters above the human sequence. **(B) **Amino acid identity between different mammalian CEACAM1 orthologues. Percent identity compared to the human sequence is given for amino acid residues comprising the beta strands of either the AA'BDE-face or the CC'C"FG-face of the immunoglobulin fold. (**C) **Schematic illustration of the proteins used in this study. Human CEACAM1-4L isoform containing a long cytoplasmic domain (hCEA1-4L), the human CEACAM1 isoform containing a short cytoplasmic domain (hCEA1-4S), and the corresponding murine isoform (mCEA1-4S) were expressed as GFP or cerulean fusion proteins. Amino-terminal Igv-like domains of CEACAM1 from human (hCEA1), mouse (mCEA1), dog (cCEA1), or cattle (isoform a, bCEA1a; isoform b, bCEA1b) were expressed in human cells as soluble GFP-fusion proteins.

The amino-terminal domain of CEACAM1 is a target for bacterial pathogens [7,8,10,23,24]. In particular, the non-glycosylated CC'C"FG-face of the immunoglobulin fold is the binding interface recognized by microorganisms [25]. To analyse if this potential evolutionary pressure by pathogens is reflected in sequence variation within this domain, we aligned and compared the published sequences of the amino-terminal immunoglobulin variable (Ig_v_)-like domain of human, murine, bovine and canine CEACAM1 (Fig 1A). Indeed, sequence differences between the mammalian species are most prominent in β-strands forming the CC'C"FG-face, whereas the glycosylated AA'BDE-face of the immunoglobulin-fold has a higher amino acid sequence identity (Fig. 1B). To test if these sequence differences result in an altered functionality with regard to pathogen binding, we generated several constructs that allowed us to test the association of CEACAM amino-terminal Ig_v_-like domains with various pathogens and to analyse their ability to mediate bacterial internalization by mammalian cells (Fig. 1C). Accordingly, we expressed Ig_v_-like amino-terminal domains derived from human, bovine, murine, or canine CEACAM1 as secreted GFP fusion proteins in human 293 cells, a cell line that does not express any CEACAM family members endogenously (Fig. 1D). Importantly, GFP-tagged fusion proteins were found in cell culture supernatants of transfected cells and were expressed at similar levels as detected by Western blotting with GFP antibodies (Fig. 1D).

### Binding of *Neisseria gonorrhoeae *to the amino-terminal domain of CEACAM1 is human specific

**Figure 2:**
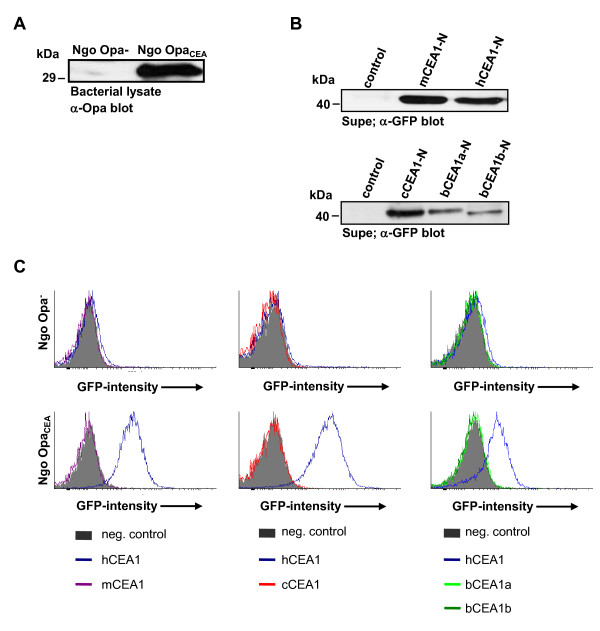
**Opa_CEA _protein expressing *Neisseria gonorrhoeae *selectively binds to human CEACAM1**. (**A) ***Neisseria gonorrhoeae *MS11 strains lacking Opa protein expression (Ngo Opa-) or expressing a CEACAM-binding Opa protein (Ngo Opa_CEA_) were lysed and the Opa protein expression was determined by Western blotting with a monoclonal anti-Opa antibody (clone 4B12/C11). **(B) **Expression of the soluble GFP-fusion proteins of CEACAM1 Igv-like domains was determined by Western blotting of culture supernatants with polyclonal anti-GFP antibody. Culture supernatants from cells transfected with a vector encoding cytoplasmically expressed GFP served as control. (**C) **Culture supernatants containing soluble GFP-tagged amino-terminal domains of the indicated mammalian CEACAMs or a control culture supernatant from GFP-transfected cells (neg. control) were incubated with Opa_CEA _protein-expressing *N. gonorrhoeae *(Ngo Opa_CEA_) or the non-opaque strain (Ngo Opa-). After washing, bacteria were analysed by flow cytometry and the bacteria-associated GFP-fluorescence was determined. Only human CEACAM1 (hCEA1) binds to Ngo Opa_CEA_.

The soluble GFP-tagged amino-terminal domains of CEACAM1 orthologues were incubated with isogenic strains of the human pathogen *N. gonorrhoeae*. The bacterial strains used either expressed a specific Opa protein, which is known to bind human CEACAM1 and other human CEACAMs (Ngo Opa_CEA_), or they did not express any Opa protein (Ngo Opa-). Opa expression by the gonococci was confirmed by Western blotting with a monoclonal antibody against neisserial Opa proteins (Fig. 2A). Following incubation with the amino-terminal CEACAM1 domains from different mammalian species, the samples were washed, and the bacteria-associated fluorescence was measured by flow cytometry. Clearly, the non-opaque bacteria (Ngo Opa-) did not reveal a positive signal in the GFP channel for any tested protein, confirming that Opa proteins are the sole neisserial factor necessary for CEACAM recognition (Fig. 2B). In contrast to the non-opaque gonococci, the Opa_CEA_-expressing bacteria clearly associated with the isolated amino-terminal Ig_v_-like domain of human CEACAM1 (Fig. 2B). Most importantly, Opa_CEA_-positive gonococci did not associate with the Ig_v_-like domains of murine, canine or bovine origin (Fig. 2B). These results demonstrate that the association of *Neisseria gonorrhoeae *with CEACAM1 is limited to the human orthologue of this protein and suggests that CEACAM1 recognition is species-specific.

### Binding of *Moraxella catarrhalis *and *Neisseria meningitidis *to the amino-terminal domain of CEACAM1 is species-specific

**Figure 3:**
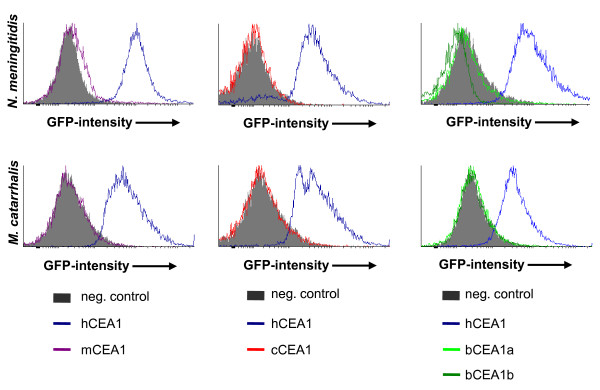
**Binding of *Neisseria meningitidis *and *Moraxella catarrhalis *to CEACAM1 orthologues**. Culture supernatants containing soluble GFP-tagged amnio-terminal domains of the indicated mammalian CEACAMs or a control culture supernatant from GFP-transfected cells (neg. control) were incubated with Opa_CEA _protein-expressing *N. meningitidis *or UspA1-expressing *M. catarrhalis*. After washing, bacteria were analysed by flow cytometry and the bacteria-associated GFP-fluorescence was determined. Only human CEACAM1 (hCEA1) binds to the pathogenic bacteria.

To extend these findings to other pathogens known to engage the amino-terminal domain of CEACAM1 we employed *N. meningitidis *MC58, a serogroup B strain, and *M. catarrhalis *ATCC 25238 in CEACAM binding assays. Accordingly, the bacteria were incubated with supernatants containing GFP-tagged amino-terminal Igv-like domains of distinct mammalian CEACAM1 orthologues, and after washing, the bacteria were analyzed by flow cytometry for associated GFP-fluorescence. Similar to what we had observed with *N. gonorrhoeae*, both bacterial species did not associate with the amino-terminal Ig_v_-like domains of bovine, murine, or canine origin (Fig. 3). In contrast, the human CEACAM1 N-terminal domain was strongly associated with both, *N. meningitidis *as well as *M. catarrhalis *(Fig. 3). These results demonstrate that several Gram-negative human pathogens selectively recognize the amino-terminal Igv-like domain of human CEACAM1 and do not bind to the same region of orthologues proteins from various mammals.

### Human, but not murine CEACAM1 mediates internalization of *Neisseria *gonorrhoeae

**Figure 4:**
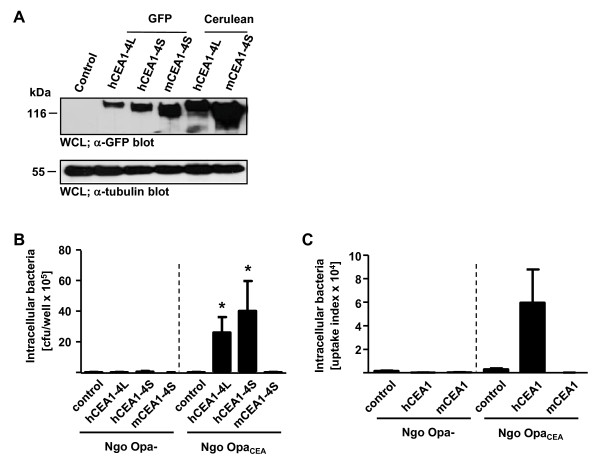
**Internalization of Opa_CEA_-expressing *Neisseria gonorrhoeae *is only mediated by human CEACAM1**. **(A) **293 cells were transfected with constructs encoding the indicated human or murine CEACAM1 isoforms fused to GFP or Cerulean. Cells transfected with a GFP-encoding vector served as control. After 48 h, cells were lysed and the expression was determined by Western blotting with a polyclonal anti-GFP antibody. (**B) **Cells transfected as in A) were infected with Opa-negative (Ngo Opa-) or Opa_CEA_-expressing *N. gonorrhoeae *(Ngo Opa_CEA_) at an MOI of 30 for 2 h. The number of viable intracellular bacteria was determined by gentamicin protection assay. Bars represent mean values ± SEM of three independent experiments done in triplicate. For statistical analysis, samples were compared against control transfected cells by one-tailed Mann-Whitney U-test; *, p < 0.001. **(C) **293 cells were transfected with constructs encoding human CEACAM1 isoform containing a short cytoplasmic domain (hCEA1), the corresponding murine isoform (mCEA1) or an empty control vector. Cells were infected with fluorescein-labelled Opa-negative (Ngo Opa-) or Opa_CEA_-expressing *N. gonorrhoeae *(Ngo Opa_CEA_) at an MOI of 30 for 2 h. The uptake index was determined by flow cytometry as described in Material and Methods. Bars represent mean values ± SEM of three independent experiments.

As the major isoforms of CEACAM1 contain 4 extracellular Ig domains, we wondered whether other determinants outside of the amino-terminal Ig_v_-like domain might influence the association with microorganisms across species boundaries. Therefore, full length murine CEACAM1-4S (encompassing four extracellular domains and the short (S) cytoplasmic domain) or human CEACAM1-4S as well as human CEACAM1-4L were expressed in 293 cells. GFP- or Cerulean-tagged human CEACAM1-4L and CEACAM1-4S, as well as murine CEACAM1-4S were expressed at comparable levels as shown by Western blotting with a polyclonal antibody against GFP, which recognizes also Cerulean (Fig. 4A).

CEACAM engagement by Opa_CEA_-expressing *N. gonorrhoeae *was evaluated through functional analysis of bacterial uptake by the transfected cells. In a first set of experiments, we used an antibiotic protection assay that is based on recovery of viable intracellular bacteria after treatment of the infected cells with gentamicin, an antibiotic that kills extracellular bacteria. In the case of non-opaque gonococci, only very low numbers of bacteria were recovered from murine or human CEACAM1-4S expressing cells similar to the numbers isolated from control transfected cells (Fig. 4B). In contrast, upon infection with Opa_CEA_-expressing *N. gonorrhoeae*, 50 - 100 times more bacteria were recovered from cells expressing human CEACAM1 (Fig. 4B). Similar to what has been observed before [18], both the short and the long isoform of human CEACAM1-4 were able to mediate efficient uptake of the pathogens (Fig. 4B). Importantly, murine CEACAM1-4S was not able to mediate internalization of Opa_CEA_-expressing *N. gonorrhoeae *consistent with the lack of bacterial binding to the Ig_v_-like amino-terminal domain of murine CEACAM1 (Fig. 4B).

To further confirm that full length murine CEACAM1-4S does not mediate bacterial internalization, we analysed transfected cells upon infection with fluorescein-labeled bacteria by an established flow cytometry method [21]. Addition of trypan blue quenches the fluorescence emitted by extracellular bacteria, resulting in cell-associated fluorescence signals derived exclusively from intracellular bacteria. In line with the results of the antibiotic protection assay, non-opaque *N. gonorrhoeae *was not internalized, whereas Opa_CEA_-expressing bacteria were taken up by cells transfected with human CEACAM1-4S (Fig. 4C). Moreover, cells expressing murine CEACAM1-4S did not harbor intracellular bacteria, further corroborating the notion that Opa_CEA _proteins of *N. gonorrhoeae *do not functionally engage CEACAM1 orthologues of other mammalian species (Fig. 4C).

### Microscopic determination of *Neisseria gonorrhoeae *internalization via CEACAM1

**Figure 5:**
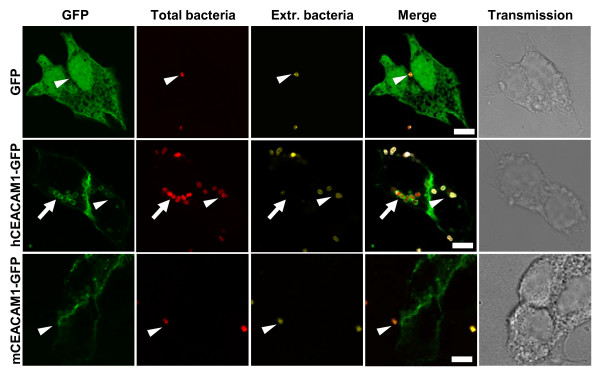
**Microscopic verification of *N. gonorrhoeae *uptake via human CEACAM1**. 293 cells were transfected with constructs encoding GFP, human CEACAM1-4S-GFP, or murine CEACAM1-4S-GFP as indicated. Cells were infected for 2 h with biotin- and rhodamine-labelled non-opaque (Ngo Opa-) or Opa_CEA_-expressing *N. gonorrhoeae *(Ngo Opa_CEA_). Infected cells were fixed, but not permeabilized, and samples were stained with AlexaFluor647-streptavidin to label extracellular bacteria (Extr. bacteria). Intracellular bacteria (small arrow) are marked by their selective rhodamine labelling, whereas extracellular bacteria (arrowheads) are stained with both rhodamine and AlexaFluor647. Bars represent 5 μm.

To finally demonstrate the selective binding and internalization of Opa_CEA_-expressing *N. gonorrhoeae *by human, but not murine CEACAM1, we analysed infected samples with confocal fluorescence microscopy. Therefore, 293 cells were transfected with GFP (negative control), human CEACAM1-4S-GFP, or murine CEACAM1-4S-GFP. Prior to infection, bacteria were labeled with rhodamine and biotin as a pre-requisite to allow the differential visualization of intracellular and extracellular bacteria [22]. Cells infected for 2 h with rhodamine/biotin-labeled bacteria were fixed and the extracellular bacteria were selectively marked with AlexaFluor647-streptavidin, which does not have access to intracellular bacteria. In GFP-expressing cells, bacteria were rarely found associated with cells (Fig. 5). Moreover, in all cases these microbes were located outside the GFP-expressing cells as evidenced by their rhodamine and AlexaFluor647 labeling (Fig. 5, arrowhead). In contrast, cells expressing human CEACAM1 contained numerous intracellular bacteria that co-localized with the GFP-tagged receptor in intracellular vesicles (Fig. 5, arrow). The absence of the AlexaFluor647 label clearly confirms the intracellular localization of these bacteria (Fig. 5, arrow). Similar to the situation in GFP-transfected cells, 293 cells expressing murine CEACAM1 showed only very few cell-associated bacteria and no intracellular bacteria were detected (Fig 5, arrowhead). Though both human as well as murine CEACAM1-4S-GFP localized on the cell surface, only human CEACAM1 is recruited to the cell associated bacteria and is co-internalized with Opa_CEA_-expressing gonococci (Fig 5). Together, these microscopic investigations provide further evidence, that only the human CEACAM1 orthologue is a target for the Opa protein adhesins of *N. gonorrhoeae *and is able to mediate the binding and uptake into eukaryotic cells.

## Discussion

Members of the CEACAM family serve as receptors for a variety of Gram-negative bacteria that live on mucosal surfaces of the human body. In an example of convergent evolution these microbes have evolved distinct CEACAM-binding adhesins that seem to promote the colonization of the mucosa. Here we provide evidence that CEACAM-binding adhesins from pathogenic *Neisseriae *and *Moraxella catarrhalis *display a high selectivity for human CEACAMs and do not associate with orthologues from non-primate mammalian species. Accordingly, the amino acid sequence divergence, that is particularly high in the CC'C"FG-face of the amino-terminal Igv-like domain of mammalian CEACAM1 orthologues, results in functional differences with regard to bacterial binding. CEACAM-binding bacterial species, which specifically colonize and infect humans, only recognize human CEACAM1 suggesting that the microbial adhesive proteins have co-evolved with their host receptor.

It has been observed earlier, that CEACAM1 orthologues from different mammalian species display high sequence diversity [4,5]. Starting from a primordial CEACAM1-like gene, CEACAMs seem to have undergone independent duplication and diversification events in different mammalian lineages resulting in an expanded family of closely related surface molecules [2,26]. Therefore, even within a mammalian order such as the primates it is difficult to assign orthologues genes except for CEACAM1 [27]. As several members of the CEACAM family are exploited by viral and bacterial pathogens, it has been suggested that the driving force behind the rapid diversification of CEACAMs in different mammalian lineages might be the selective pressure by pathogens [3,28].

An additional example of CEACAM1 recognition by pathogens is found in rodents, where the mouse hepatitis virus strain A59 (MHV-A59), belonging to the coronavirus complex, binds via its spike protein to murine CEACAM1 [29,30]. Of the two CEACAM1 alleles present in the mouse population, MHV-A59 selectively recognizes CEACAM1a and only marginally binds to the CEACAM1b allele [31]. Therefore, inbred mouse lines that carry the CEACAM1a allele are susceptible, whereas lines carrying the CEACAM1b allele or CEACAM1-deficient mice are resistant to MHV-A59 [32]. However, despite this selectivity for the murine CEACAM1a allele, it has been shown that several MHV strains, including A59 and MHV-2, can utilize human CEACAM1 as well as CEA to infect eukaryotic cells in vitro [33].

In contrast to this promiscuity of host receptor utilization, our results highlight the specificity of bacterial adhesins for human CEACAMs. Consistent with the strict selectivity of these pathogens for humans as natural host organisms, they only associate with human CEACAM1. Accordingly, the bacteria can efficiently invade only cells that express the human orthologue of CEACAM1, but not the murine orthologue. It is interesting to note, that additional pathogenicity factors of these bacteria show a similar exquisite specialisation for human molecules. For example, the neisserial IgA1 protease [34] only cleaves human IgA1 molecules, but not IgA molecules from other mammalian species. Similarly, the transferrin-binding protein, that is critical for iron acquisition in the human host, can utilize only transferrin from human sources or from closely related apes such as chimpanzee [35,36]. Gonococci are also able to escape from host complement attack by recruiting complement component 4b-binding protein (C4bp) [37]. This ability is again specific for human C4bp and even chimpanzee C4bp only provides protection from complement for some, but not all gonococcal strains [38].

Besides immune escape and nutrient acquisition, our results reveal another area, where these Gram-negative pathogens employ species-specific pathogenicity factors. Clearly, adhesion to the mucosal surface epithelium is the initial step in the colonization by CEACAM-binding bacteria, and the possession of adhesive proteins specifically targeting human CEACAMs might promote this step. However, at the same time this specialization could contribute to the limited host spectrum not only of pathogenic Neisseriae, but also of *M. catarrhalis *and *Haemophilus influenzae*.

## Conclusions

Recognition of host surface structures is critical for many bacterial pathogens to establish a first foothold in their target organism. Whereas a high degree of specificity might allow intimate binding of the microorganisms to eukaryotic cells, it might at the same time limit the host range of the pathogen. Here we reveal a selective interaction between bacteria and the human form of the cell surface receptor CEACAM1 that correlates with the human-restricted pathogenicity of these microbes. Our analysis not only points to an ongoing pathogen-host co-evolution at the level of receptor-adhesin interaction, but further strengthens the idea that the Opa_CEA _protein-mediated interaction with human CEACAMs might provide an access point for preventing or limiting infection.

## Authors' contributions

CRH, MV, UG, and RK conceived of the study, MV and CRH designed the experiments, MV and VB performed the experiments, CRH and MV wrote the paper. All authors read and approved the final manuscript.
